# What do patients experience? Interprofessional collaborative practice for chronic conditions in primary care: an integrative review

**DOI:** 10.1186/s12875-021-01595-6

**Published:** 2022-01-14

**Authors:** Alexandra R. Davidson, Jaimon Kelly, Lauren Ball, Mark Morgan, Dianne P. Reidlinger

**Affiliations:** 1 Faculty of Health Sciences and Medicine, Bond University, Gold Coast, Australia; 2 Centre for Online Health, Faculty of Medicine, The University of Queensland, Brisbane, Queensland Australia; 3 Centre for Health Services Research, Faculty of Medicine, The University of Queensland, Brisbane, Queensland Australia; 4 Menzies Health Institute Queensland, Griffith University, Gold Coast, Australia

**Keywords:** Collaboration, Primary health care, Interprofessional collaboration, Chronic conditions, Patient-centered care

## Abstract

**Background:**

Improving the patient experience is one of the quadruple aims of healthcare. Therefore, understanding patient experiences and perceptions of healthcare interactions is paramount to quality improvement. This integrative review aimed to explore how patients with chronic conditions experience Interprofessional Collaborative Practice in primary care.

**Methods:**

An integrative review was conducted to comprehensively synthesize primary studies that used qualitative, quantitative, and mixed methods. Databases searched were Medline, Embase, CINAHL and Web of Science on June 1st, 2021. Eligible studies were empirical full-text studies in primary care that reported experiences or perceptions of Interprofessional Collaborative Practice by adult patients with a chronic condition, in any language published in any year. Quality appraisal was conducted on included studies using the Mixed Method Appraisal Tool. Data on patients’ experiences and perceptions of Interprofessional Collaborative Practice in primary care were extracted, and findings were thematically analyzed through a meta-synthesis.

**Results:**

Forty-eight (*n* = 48) studies met the inclusion criteria with a total of *n* = 3803 participants. Study quality of individual studies was limited by study design, incomplete reporting, and the potential for positive publication bias. Three themes and their sub-themes were developed inductively: (1) Interacting with Healthcare Teams, subthemes: widening the network, connecting with professionals, looking beyond the condition, and overcoming chronic condition collectively; (2) Valuing Convenient Healthcare, subthemes: sharing space and time, care planning creates structure, coordinating care, valuing the general practitioner role, and affording healthcare; (3) Engaging Self-care, subthemes: engaging passively is circumstantial, and, engaging actively and leading care.

**Conclusions:**

Patients overwhelmingly had positive experiences of Interprofessional Collaborative Practice, signaling it is appropriate for chronic condition management in primary care. The patient role in managing their chronic condition was closely linked to their experience. Future studies should investigate how the patient role impacts the experience of patients, carers, and health professionals in this context.

**Systematic review registration:**

PROSPERO: CRD42020156536.

**Supplementary Information:**

The online version contains supplementary material available at 10.1186/s12875-021-01595-6.

## Background

Interprofessional Collaborative Practice (IPCP) is recognized as an essential component of high-quality healthcare. The World Health Organization (WHO) describes collaborative practice as occurring when multiple health workers from diverse professional backgrounds work with patients and their families to deliver high quality care and interventions [1]. The involvement of patients as central to healthcare is now recognised as essential, and differs fundamentally to traditional clinician-centered healthcare [2]. In the primary care setting, IPCP teams are encouraged to involve patients and their support persons as members of the team [2].

Teams of primary health care professionals, including general practitioners (GPs), practice nurses and allied health providers working with patients with chronic and multimorbid conditions have been shown to improve health-related outcomes and costs in primary and community settings [3]. However, it has been suggested that IPCP in a primary care context is more challenging to implement than other healthcare settings [4]. In comparison to the hospital setting, IPCP in primary care is less well defined and structured [5, 6]. Team characteristics and team processes such as transparent communication, role clarification and co-location have a strong influence on patient healthcare goals [7]. Barriers to IPCP in primary care include off-site location of allied health services, lack of formal team structures and leadership, the absence of a common vision and goals, and poorly defined roles [8, 9].

Interprofessional collaboration and IPCP has been researched extensively, including in the primary care setting. Previous review papers have explored if and how IPCP meets several aspects of the quadruple aim of healthcare [10], including health-related outcomes [11], healthcare costs [7] and provider experience and perspectives [4, 12, 13]. Specifically, the literature is dominated by primary healthcare professional views and experiences [12, 13]. Of the quadruple aim of healthcare, the patient experience and voice has been researched the least and is pertinent to healthcare improvement and thus worthy of investigation.

A 2020 scoping review aimed to identify studies that explored the patients’ perspective of IPCP in primary care [14]. However, the review has several limitations that differ from this integrative review, including a restricted publication year limit of 1997–2017, only including studies published in English, a single database was used for the search and patient populations were not restricted to chronic conditions. The scoping review identified seven papers for inclusion, and the authors recommended further exploration into the topic of patient experience of IPCP [14].

The quadruple aim of health care explicitly lays out an approach to health service improvement which includes the aim to improve patient experience of care, improve the health of populations, reduce healthcare-related costs, and improve health provider experience [10, 15]. In the primary care setting, a review of health professionals’ perspectives reported on the facilitators and barriers regarding IPCP [16]. What is not yet known is how patients themselves experience IPCP in primary care, including their perceived role in the collaboration, and how IPCP enhances the safety, quality, and outcomes of the care they receive. This integrative review aims to understand how patients with chronic conditions experience interprofessional collaborative practice in the primary care setting.

## Methods

An integrative review design was used to synthesize literature on patients’ experiences of IPCP. An integrative review allows for comprehensive synthesis of findings from quantitative, qualitative and mixed methods study designs, to assist understanding a phenomenon of interest [17]. This method was selected as most appropriate to understand the experience of patients with chronic conditions in IPCP in primary care as it enabled a deeper analysis by including diverse study designs and has been used for similar topics in this area of healthcare previously [4, 6]. The five steps outlined by Whittemore and Knafl were used to guide the review process: problem identification, systematic literature search, data evaluation, data analysis, and presentation [17].

The integrative review protocol was registered with PROSPERO: CRD42020156536, and this manuscript is written in accordance with the Preferred Reporting Items for Systematic Reviews and Meta-Analyses (PRISMA) checklist (see Additional file 1) [18].

### Sample and inclusion/exclusion criteria

We sought to answer the primary question ‘How do individuals with a chronic condition experience IPCP in primary care?’ using the SPIDER (sample, phenomenon of interest, design, evaluation, research type) tool [19]. SPIDER criteria were: Sample, individuals with a chronic condition that has lasted, or is expected to last, for a minimum of 6 months [20]; Phenomenon of Interest, interprofessional collaboration in the primary care context; Design, all study designs; Evaluation, experiences or perspectives or views or attitudes or feelings or perceptions; and Research Type, original primary studies. Inclusion criteria was: (i) empirical full-text studies, (ii) conducted in the primary care setting, (iii) reporting experiences or perceptions of interprofessional collaborative practice by patients with a chronic condition, (iv) any language, (v) adult participants, and (vi) published in any year.

Non-English studies which were deemed potentially eligible were translated into English by an individual who was bilingual in English and the language of the study, and with a minimum of a bachelor’s education level. Studies that were conducted in, or recruited patients from, a hospital inpatient or outpatient setting were excluded. Further, studies where participants were solely health professionals, or where patient experiences were inseparable from those of health professionals, were excluded.

### Literature search

An initial systematic literature search was conducted on June 1st, 2021. A three-step approach to searching for original published peer-reviewed articles was conducted. In step one a search of MEDLINE and CINAHL was conducted using search terms outlined in Table 1 and the SPIDER, followed by an analysis of key words in the title and abstract and or the index terms used to describe the article. In step two a second, comprehensive search using all identified keywords and index terms was then conducted across four electronic databases: MEDLINE, CINAHL, Embase and Web of Science. The MEDLINE search strategy is available in Additional file 2. In step three handsearching was conducted of the reference list of all included full-text articles for any additional articles.

**Table 1:** Search Terms Example

Population	Context-Interprofessional Collaborative Practice	Setting -Primary care	Outcome
Patient Consumer Client	Interprofessional Multidisciplinary Interdisciplinary Collaboration Teamwork	General Practice Private practice Community Practice Ambulatory Practice	Experience Perception Perceive Perspective View

Due to the lack of consensus on key terminology used for IPCP [6, 21], healthcare models reflective of IPCP and terms commonly used to refer to such models in the literature (as identified in step one of the search strategy) were used in step two. For example, “case management”, “collaborative care”, “coordinated care”, “chronic care model”, “medical home”, “healthcare home”, “team care arrangements”, “care planning”, “patient health record”, “team care”, and “multidisciplinary care” were used in the search. In addition, database-specific key words and subject headings were also used, for example, MeSH terms for MEDLINE. The literature search was developed and run with the input of an experienced health sciences and medicine librarian.

Endnote X9 [22] and Covidence [23] were used to manage citations during screening and selection. All records were independently screened by title and abstract against the predetermined inclusion criteria by two researchers. For those results not excluded at title and abstract stage, the full texts were independently screened against the inclusion criteria by the primary researcher (AD) and a second researcher drawn from the team of co-researchers. Excluded studies were grouped according to the reason for exclusion (e.g., setting, population, not full text). Any disagreements that arose during screening and selection were resolved through discussion or consultation with a third researcher.

### Data extraction and evaluation

Data were extracted by one researcher, and checked by a second researcher, using a table developed by the research team including author, year, country, aim, research design, sample, participants, chronic conditions(s), and key findings that related to patient experience of IPCP. Critical appraisal of the data was conducted by two independent researchers using the Mixed Methods Appraisal Tool (MMAT), version 2018 [24]. Discrepancies between scores were discussed between the two researchers. When consensus could not be reached, a third researcher was consulted. MMAT was used as it allows critical appraisal of all included study methods: qualitative, quantitative, and mixed methods.

### Data analysis

Qualitative, quantitative and mixed methods studies were analyzed thematically using the five-stage meta-synthesis process outlined by Whittemore and Knafl: 1) data reduction, 2) data display, 3) data comparison, 4) conclusion drawing and 5) verification [17]. The outcome of meta-synthesis in an integrative review is an integrative interpretation of results to offer a novel finding. The meta-synthesis process was underpinned by the research paradigm of constructivism, an approach to research that acknowledges the different elements that individuals draw on to make meaning of their lived experience [25]. Studies underwent iterative reading and comparison, to cluster recurring themes and sub-themes. Findings from each study were independently read, coded, and organized into categories. Codes and categories were compared across studies to identify patterns, relationships, and themes. Draft themes, sub-themes and exemplar quotes were developed and discussed amongst all researchers before finalization. In this review, to communicate the lived experience of individuals within each study, the meta-synthesis results were presented from the perspective of patients.

## Results

**Fig. 1:**
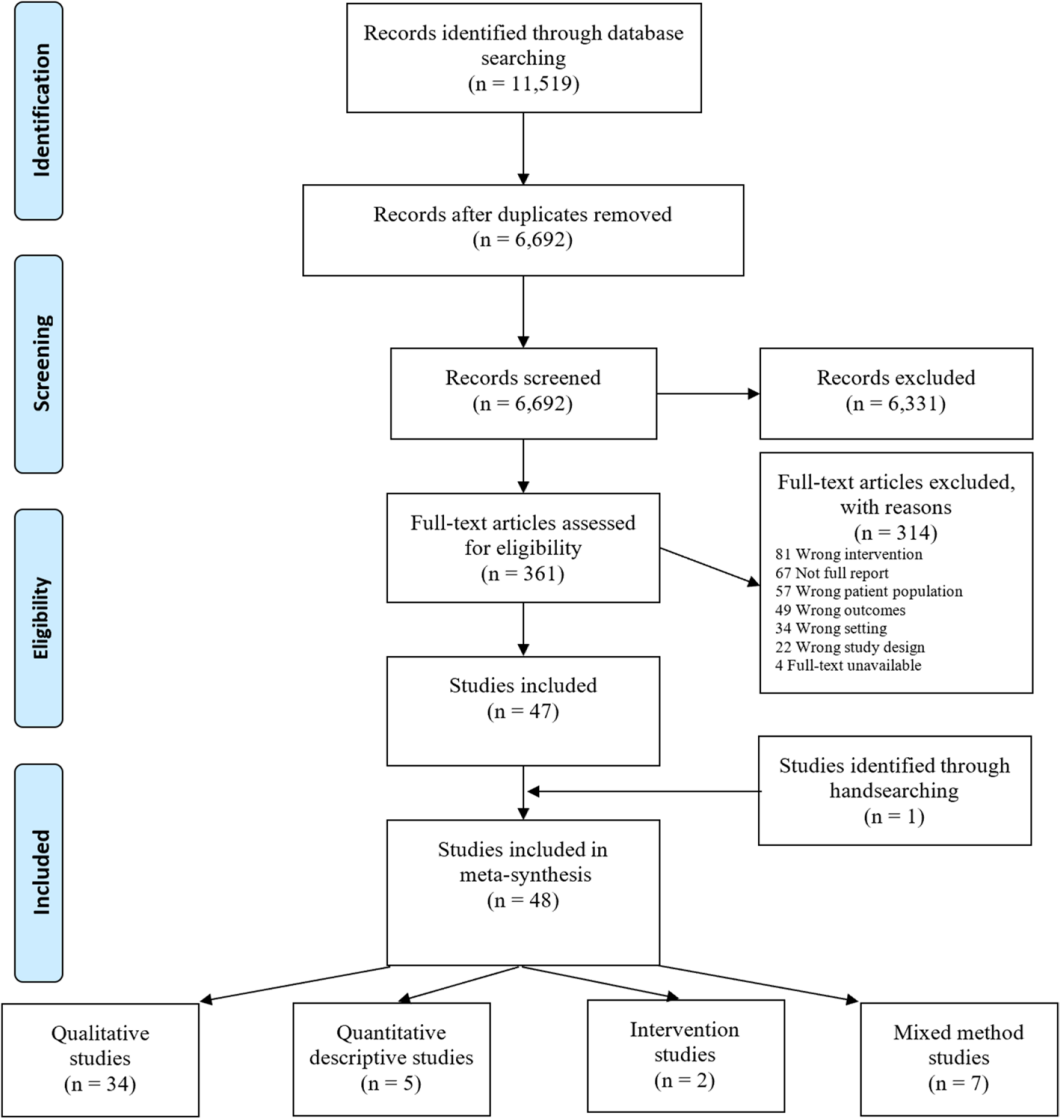
PRISMA flow diagram of study selection process

The PRISMA [18] flow diagram (Fig. 1) shows the search retrieved 6692 articles after de-duplication; 47 studies met inclusion criteria and one additional study was identified through hand-searching of reference lists. Studies used a range of methodological designs, including qualitative focus groups and/or interviews (*n* = 34) [9, 26–58], mixed method studies (*n* = 7) [59–65], quantitative descriptive studies (*n* = 4) [66–69], and intervention studies (*n* = 2) [70–72]. Across studies there was a total of *n* = 3803 participants, including patients, informal carers and family members. Studies were mostly conducted in the USA (*n* = 13) [29, 40, 42, 44, 54, 55, 59, 61, 65, 67–69, 72], Australia (*n* = 11) [26–28, 30, 34, 36, 41, 52, 57, 63, 66], UK (*n* = 7) [31, 39, 46–48, 53, 60], Sweden (*n* = 4) [38, 49–51], Canada (*n* = 3) [32, 35, 45] and The Netherlands (*n* = 2) [9, 64] with one study undertaken each in Belgium [62], Italy [71], France [33], New Zealand [43], Norway [58], Qatar [56] and, Spain [70], and one study conducted across multiple European countries [37]. Key characteristics of included studies are outlined in Additional file 3.

The conditions covered in the studies were: diabetes type 1 and type 2 (*n* = 14), mental health conditions such as depression and anxiety (*n* = 9), chronic pain (*n* = 4), cardiovascular conditions including chronic health failure (*n* = 3), HIV/AIDs (*n* = 2), asthma (*n* = 1), dementia (*n* = 1), cancer (*n* = 1), with the remaining studies examining a range of other conditions or multimorbidity (*n* = 13).

The methodological quality of studies varied according to the MMAT outlined in Additional file 4. Common limitations in the qualitative studies were an unclear explanation for the selected methodological approach [26, 32, 37, 41, 43, 66], findings were inadequately derived from the data [36, 42, 47, 52, 55], or supported by the data including too few quotes to support thematic analysis [33, 36, 41–43, 47, 48, 52, 55, 57], and a lack of coherence between qualitative data, collection, analysis and interpretation [36, 41, 47, 48, 54, 57, 58]. Limitations for the single randomized-controlled trial was the low response rate of < 50% and that the randomization was inadequately described in the report [72]. Common limitations for quantitative non-randomized studies (one cross-sectional and one cohort study) were a lack of accountability of confounders [70, 71]. Limitations for quantitative descriptive studies (*n* = 3 surveys) included the sample not representing the target population [66–68], and that the risk of nonresponse bias was high [66–68]. Adequate explanation of divergence between quantitative and qualitative data [60, 62–65] and adherence to quality criteria for each tradition of methods included [60–65] were the most common limitation for mixed method studies.

### Meta-synthesis

Three themes were inductively developed: (1) Interacting with Healthcare Teams, (2) Valuing Convenient Healthcare and (3) Engaging in Self-care. Each theme is supported by four, five and two sub-themes, respectively.

### Interacting with health care teams

Within this theme, participants described their key experiences interacting with healthcare teams as represented across four sub-themes: (1) Widening the network, (2) Connecting with professionals, (3) Looking beyond the condition, and (4) Overcoming chronic condition collectively.

#### Widening the network

Participants described how their network widened through IPCP to include professionals beyond their GP such as nursing, allied health, specialists, community and social services, and family/friends/carers. This widening enabled gaps in “traditional” or “usual care” by a sole practitioner, typically a GP, to be filled by other team members:

*“I’m in a win-win situation. There’s no way I’d get the care and attention from [the GP] who’s very busy that [the nurse] can give me… I’m the benefit, a recipient of teamwork.”* – Patient, Pullon 2011 (CarePlus Programme – teamwork intervention with GPs and nurses, General Practice, New Zealand, multiple conditions).

#### Connecting with professionals

Participants described how they connected with individual professionals in their network. Describing a relationship and connection with the team, participants found the care to be a more positive experience. Participants who described motivation, ability and drive to self-advocate were more likely to perceive a stronger connection with the team than those who did not. Teams that were described as supportive and trust-worthy, participants were willing to engage with:

*“What kept me coming was people telling me I was doing good [sic], they were proud of me. I really felt loved when I did not love myself. Staff are great; I’m treated with respect and like a human being.”* – Participant, Drainoni 2014 (Substance Abuse and HIV treatment and prevention program with physician, nurse and addictions counsellor, USA, health insurance co-payment, HIV, and substance use disorders).

Participants connected with professionals via multiple communication modes: face-to-face, telephone, and email. Participants appreciated the greater access to professionals, such as when they had a question, but did not feel it warranted a consultation. Participants felt comforted, knowing they had this connection and communication readily available:

*“The fact they’ve come home personally to you and got in touch by telephone every week has led to some sense of security.”* – Patient, Talabani 2017 (Palliative advanced homecare, Sweden, Heart Failure)

#### Looking beyond the condition

Participants described how they received and experienced holistic care incorporating physical, mental, social, and spiritual wellbeing. Integration of different backgrounds, including medical, behavioral, and complementary practitioners, contributed to this holistic care experience. Feeling treated as both a patient and a person was described as holistic care:

*“They see me as a whole person; you can talk to them about almost anything.”* – Patient, Talabani 2017 (Palliative advanced homecare, Sweden, Heart Failure)

In some cases, participants requested more holistic care, but felt care was diagnosis-focused and did not incorporate their broader needs:

*“[The diabetes specialist GP] was more concerned about my diabetes than my overall wellbeing… Diabetes is a complex condition where you have to treat the individual on multiple levels. …You have to know what’s going on in that person’s life and their life-style, because their management has a lot to do with their responsibilities – employment, children, their overall outlook...”* – Patient, Burridge 2017 (GP-led multidisciplinary team, General Practice, Australia, T2DM).

#### Overcoming chronic condition collectively

Participants described their health networks as taking a collective approach to managing and overcoming physical and social challenges associated with living with a chronic condition. Patient healthcare networks working collectively was consistently reported across studies, where participants described more positive experiences when network members were on the same page:

*“… Connected physical and mental symptoms and team worked together on the same page.”* – Long-term intervention group ranked higher than control and short-term intervention group, Reiss-Brennan 2014 (Mental Health Integration in Primary care with control vs short- and long-term intervention, Primary Care, USA, Depression).

### Valuing convenient healthcare

This theme represented participants’ experiences with team structures that influenced the convenience of their healthcare including co-location of professionals and services, multidisciplinary appointments, length of appointments, care accessibility, care plans, a care coordinator, and the affordability of health care. These are reflected in the five sub-themes: (1) Sharing space and time, (2) Care planning creates structure, (3) Coordinating care, (4) Valuing the role of General Practitioners, and (5) Affording healthcare.

#### Sharing space and time

Participants described that the care provided by co-located professionals better reflected team care, including improved communication, teamwork, availability of services such as pharmacy, x-ray, mental and allied health.

*“…it’s not just a minor health centre where you go and see the GP for minor ailments and things like that but there are other treatments and things available where you can be interviewed for various other things besides just ordinary medical issues, like physiotherapies and things like that… you have a chemist available in the building and the X-ray facilities are also integrated into the building as well…”* – Consumer, Banfield 2017 (Inclusion of allied health in General Practice team care, Australia, Government-funded Medicare scheme CDM, multiple chronic conditions).

Participants also described varying experiences on length of appointments with professionals. The longer the appointment, the more a patient felt listened to and able to communicate their issues. Participants highlighted the GP as time-poor and unable to provide the same in-depth conversation that other less time-poor professionals could, such as nurses, allied health, and community health workers (CHWs):

*“…Thanks to all the CHWs that take the time to explain and to answer questions, I don’t feel they do things in a hurry like other programmes. I like the way they treat me.”*– Patient, Otero-Sabogal 2010 (Team-based self-management intervention with CHW, Primary Health Clinic, USA, T2DM).

#### Care planning creates structure

Participants described formal care plans provided structure and equated to positive care experiences. Studies that investigated participants’ experiences with care plans found that they rated their care as high or described better overall experiences. Beneficial aspects of the care plan were having a clear outline, improved or initiated access to allied health, description of who was involved and their role, when follow-up would occur, and enabled self-management.

Patients described that when care plans were reflective of their personal goals and care needs, the experience was positive:

*“They found out what my life is about and what is important to me. I got to decide what I really wanted to work on, kind of what my goals were… It was tailored for me.”* – Veteran Patient, Purcell 2019 (Interdisciplinary, integrated pain teams, Primary VA Medical Centers, USA, Chronic pain, and opioid misuse)

Not all experiences were positive. For example, with mental health care plans, where participants felt that although plans provided access to psychological services, the care received was not beneficial, and there was room for improvement:

*“It is instrumentally useful in enabling me to access cheaper psychology services. Otherwise, it has never really helped. They are one off items. What people need are high quality ongoing medical and allied services. The plans do not enable adequate, let alone high quality, mental health services to arise from the ether. Until such services are created the plans remain a bureaucratic exercise for many, probably for most with significant mental illness.”* – Mental Health Participant, Banfield 2019 (Medicare-funded Mental Health Care Plan between GP and psychologists, Australia, mental health conditions).

#### Coordinating care

Participants described care coordination occurring within their healthcare network. Care coordination was positive when they had a central care coordinator, who could have been their GP, nurse, occupational therapist, diabetes educator, counsellor, or mental health worker. Participants outlined benefits of having a care coordinator, including having a key contact person, a hub for medical information and notes, and someone to guide and lead them in their care when they were unable to. Overall, participants reported positive experiences with various health professionals acting in a coordinator role; what was vital was that the care coordinator was present.

Many patients commented that their GP was a problem-solver, and some preferred their GP as coordinator despite being time-poor, as they perceived their GP to be the most knowledgeable professional, for example:

*“Once you’ve got a doctor tell you what to do and then prescribe your tablets, if something is not working, you’re talking to your doctor…”* – Patient, Tan 2013 (Extended pharmacist services integrated in GP practices, General Practice, Australia, multiple conditions).

Patients identified that the nurse was someone they could confidently ask questions to, for example:

*“It sounded like a good idea to have somebody else [the nurse] in there that you could call and talk to and ask questions and then she would find the answer and get back to us.”* – Patient, Wilson 2018 (Chronic Care Management services including a team of physicians, nurse practitioners, physician assistants, clinical nurse, and nurse midwives, USA, health insurance co-payment, multimorbidity).

Other health professionals were outlined as coordinators, such as the diabetes educator:

*“My first interview was with the educator and she worked it all out for me, a dietitian and everything, and she worked out everything for that…”* – Patient, Hepworth 2013 (MDT including endocrinologist, GP, diabetes educator and podiatrist, Integrated Primary Care, General Practice, Australia, T2DM).

#### Valuing the general practitioner role

Despite being time-poor, participants described their GP as crucial in their care because of the GP’s care coordinator role. When challenges or changes to team-care arose, participants’ satisfaction was unchanged because of their strong GP relationship, described as being built on trust. Rapport with their GP enabled participants to voice their opinions about their care, including taking on a more active role providing feedback on care decisions. Where participants perceived the GP had a poor attitude, this hindered open communication. The relationship with their GP influenced their perceptions of other aspects of care. If participants had a positive relationship with their GP, the overall care experience was also positive, including their relationship with other team members:

*“…based on long experience with certain doctors here [laughs] we just mentioned that we’ve known [doctor] for 35 years plus and some of his staff are also here now. I have full confidence in the resources of [healthcare centre] …the same people and the same good care.”* – Consumer, Banfield 2017 (Inclusion of allied health in General Practice team care, Australia, Government-funded Medicare scheme CDM, multiple chronic conditions).

#### Affording healthcare

Participants had overall positive experiences of subsidized health professional appointments or healthcare programs, whilst insurance funds were still available. Some experienced affordability issues when initiating or continuing treatment after external subsidies or incentives had ceased. Individuals of lower socioeconomic status and pensioners experienced more significant struggles to afford team healthcare services. Because patients were unable to afford care, this then led to the necessity to find money through reducing funds in other areas of life or discontinuation of care. Additionally, participants with mental health conditions found the discontinuation of healthcare services due to funding was detrimental:

*“We really, really hit it off, but then when my insurance wouldn’t cover it, it really took a toll on me cause I like to keep the same counsellor. It’s really hard to switch… it’s really, really hard to open up to somebody new.”* – Patient, Davis 2018 (Private, not-for-profit Community Mental Health Centre, integrated medical and behavioral health professionals, in rural USA, private health insurance, mental health conditions)

### Engaging in self-care

Participants described approaches to engaging in their care as either passive or active. Engaging in their healthcare, whether by invitation or self-initiation, was perceived to be beneficial by patients. They experienced shared decision making, being informed in their own treatment/management, and partnering with the team.

#### Engaging passively is circumstantial

Participants experienced professionals as accommodating the care and plans to suit their needs. For these people, care planning was described as having little input from the person, and this was adequate as they trusted the professionals to make decisions on their behalf. “Depending on individual circumstances, such as reduced capacity to care for themselves due to frailty, their age or their condition, and the view and trust that professional-led care produced desirable outcomes, some patients appreciated this guidance from professionals:”

*“To go along with what the doctors and nurses are saying… I think that is very important. I’m quite happy with [the new care].”* - Patient, Burridge 2016 (GP-led multidisciplinary team, General Practice, Australia, T2DM).

#### Engaging actively and leading care

When participants perceived that care provided was not meeting their needs, they took a proactive approach to lead care. Centering themselves in care included wanting to participate in IPCP team discussions and meetings, perceived as their ‘right’. In some cases, such as asthma, participants perceived they were responsible for liaising between professionals, and if they were well-informed, they did not mind if professionals were not informed and did not liaise with one another:

*“For me, [the communication between physicians] it does not matter if it returns or it does not return. In fact, it’s me who takes care of my health. So as long as I’m informed… Let’s say that I am the person responsible.”* – Patient, Hannane 2019 (GPs and nurses collaborating, General Practice, Asthma, France)

Participants expressed motivation to take a proactive approach to be involved in their health care and be an active part of their IPCP team:

*“I’m being optimistic, so I’m not just going to forget, I’m not just going to give up. I’m going to try… I’m going to try my hardest and hopefully...”* – Patient, Fu 2018 (Team of nurses, physiotherapists, healthcare trainers and doctors, Public Chronic Pain Clinic, UK, Chronic Pain).

## Discussion

This integrative review aimed to explore how patients with chronic conditions experience IPCP in primary care resulting in 48 studies meeting inclusion criteria, which was more comprehensive than a scoping review published in 2020 [14]. A better understanding of the patient experience of this model of care is important as patient experience has a profound effect on clinical outcomes and is a key healthcare outcome measure [10, 73]. The themes reflect overall patients were positive about their IPCP experiences in primary care. Key experiences also highlighted patient-perceived limitations with the IPCP model of care. Some patients described a lack of clarity about their role in team care, discontinuation of care once funding of programs ceased, the view that increased access to professionals did not necessarily increase care quality and, inconsistent holistic care within the IPCP model.

Clarification and definition of roles within healthcare teams is a crucial component of IPCP [1, 74]. The ‘Engaging in Self-care’ theme highlighted a large divide in how patients engaged with the healthcare system, including professionals. The level of engagement or role that patients took in their healthcare was variable between and within studies. This variability was largely because studies aimed to explore overall experiences but did not focus on the patient role to engage with IPCP teams. The role of health professionals in IPCP has been well explored in the healthcare literature, from the perspective of professionals [8] and patients [75]. However, the patient role in IPCP is still relatively unclear. A key component of the WHO IPCP framework is role definition leading to better understanding of the IPCP team structure [1]. Frameworks for IPCP such as the one developed by the WHO, do not provide guidance on how health professionals should engage patients in IPCP, or what roles patients could or should play. Individual patient roles of self-care in IPCP could be supported by incorporating an understanding of supports external to formal healthcare, such as social networks and relationships [76]. Since the definition of IPCP includes health professionals working with patients, family, carers, and communities to provide high quality healthcare, knowing whether and how patients engage is therefore essential to effective IPCP [1].

Funding models that support continuity in care and provide high quality healthcare are a priority in chronic disease management in primary settings [77]. A key limitation of IPCP was outlined in the ‘Valuing Convenient Healthcare’ theme, the sub-theme ‘Affording healthcare’ demonstrated how patients experienced a lack of continuity of care when funding for IPCP care ceased. Healthcare funding models, such as Medicare in Australia or private health insurance in USA [78], enable access to IPCP services. However, there is limited care continuity in services when funding ceases from the perspectives of providers [79] as well as patients, as expressed by studies in this review. In addition, co-payments have been linked to a decrease in usage of healthcare as individuals cannot or do not wish to pay an out-of-pocket cost alongside the Government incentive or private health coverage [78]. Continuation of care with sufficient healthcare funding is a global issue at all levels of primary healthcare: community-level, service-level and system-level [80].

Including patients in the development of care plans can have positive impacts on healthcare outcomes [81, 82]. Divergence within the ‘Valuing Convenient Healthcare’ theme was evident within the sub-theme ‘Care Planning Creates Structure. On one hand, when care plans were devised in consultation with the patient, participants described this as a positive experience. On the other hand, patients perceived that although care plans improved access to other health professionals, they did not always experience an improvement in their care. Care plans for specific patient populations have demonstrated positive impacts, such as Cancer Survivorship Care Plans in providing patients with information on their care trajectory [81]. However, care plans alone did not influence distal outcomes such as patient-reported health status, perceptions of care, health care delivery, and health care use [81]. A systematic review on Personal Asthma Action Plans and their impact on healthcare outcomes concluded that the best results were seen when individuals had ownership and understanding of the action plan [82]. Since care plans have been described as a facilitator to IPCP previously [83], they should therefore be used in management of chronic conditions in primary care.

Patients’ desire for holistic care and to be treated as a person rather than as a patient is consistent across all healthcare settings [84]. A further key finding, highlighted within the ‘Interacting with Healthcare Teams’ theme, was captured within the sub-theme ‘Looking beyond the condition’. Experiences were divergent within this sub-theme, with some studies highlighting participants felt their care was holistic and looked beyond their condition, whilst other studies demonstrated the opposite. This is consistent with findings described in other literature across other settings, for example, inpatient mental health, where being treated as a person was valued by participants [85]; outpatient dialysis clinic for end-stage kidney disease, where holistic care needed to be better incorporated for decision-making [86]; and an inpatient rheumatoid rehabilitation program, where participants felt seen as a whole person [87]. This indicates that, across different settings and conditions, patients consistently want to be seen as a person rather than the disease they have been labelled with.

### Strengths & Limitations

This systematic review has several strengths including the inclusion of all study designs to obtain the patient experience assessed from different methods, no year or language limit resulting in an additional six non-English papers being included after translation, duplicate screening, and quality assessment methods. Despite the inclusion of studies with no language limit, there was unclear and minimal representation of minority groups in the included original studies, including Indigenous, LGBTQIA+ and culturally and linguistically diverse backgrounds. Future research into this field could incorporate these groups to obtain their views and experiences. A key limitation was the narrow inclusion criteria, which restricted studies to those that reported on patient views; however, those reporting only the views of family, friends and carers were omitted which could overlook vital viewpoints on IPCP. Another key limitation was the overall relative low quality of studies, and the inclusion of studies that did not have the patient experience as a primary aim but had apparently been included as an ‘afterthought’ [40, 65, 71]. Additionally, there is potential for positive publication bias of the included original research studies. There were dissenting views in the included studies, however the overwhelming positive experiences could be due to publication bias based on the direction of results [88].

## Conclusion

The overall positive experiences of patients of IPCP demonstrates that this model of care is valued and is appropriate for chronic conditions in primary care. The key experiences indicating areas of potential improvement should be explored further to identify improvement strategies. Improvement strategies could focus on supplementary funding for IPCP, closing the disparity between care plan intentions and practice use of plans, consistently holistic interventions, and clarification of the patient role in the IPCP model. Thus, further studies should seek to explore these potential improvements to improve patient experience. In particular, what roles patients currently play and whether this role could change to improve healthcare experiences and other healthcare outcomes. Since patients, their family and carers, and health professionals were identified as the key players in IPCP in primary care, it is recommended that the exploration of the patient role in IPCP come from these three perspectives.

## Supplementary Information

**Additional file 1.** PRISMA 2020 Checklist.

**Additional file 2.** MEDLINE Search Strategy.

**Additional file 3. **Description of Included Studies.

**Additional file 4.** Mixed Method Appraisal Tool of Included Studies.

## Data Availability

Not applicable as there is no original data in this manuscript.

## Abbreviations

CHW: Community Health Worker
IPCP: Interprofessional Collaborative Practice
GP: General Practitioner
MMAT: Mixed Methods Appraisal Tool
PRISMA: Preferred Reporting Items for Systematic Reviews and Meta-Analyses
SPIDER: Sample, phenomenon of interest, design, evaluation, research type
WHO: World Health Organization
