# Supplementary material for: What do patients experience? Interprofessional collaborative practice for chronic conditions in primary care: an integrative review
Source: BMC Prim Care. 2022 Jan 14;23:8. doi: 10.1186/s12875-021-01595-6 (PMC8759162; doi:10.1186/s12875-021-01595-6)
Supplement: Supplementary file 2 — Additional file 2. MEDLINE Search Strategy. [file 12875_2021_1595_MOESM2_ESM.docx]

MEDLINE Search Strategy Ran on 1^st^ of June 2021 for project titled:

Davidson et al. 2021 “What do patients experience? Interprofessional collaborative practice for chronic conditions in primary care. An Integrative Review.”

(Interdisciplin*.ti,ab. OR Multidisciplin*.ti,ab. OR Interprofession*.ti,ab. OR Inter-profession*.ti,ab. OR Case Manage*.ti,ab. OR Care Manage*.ti,ab. OR Interprofessional Relations/ OR Interdisciplinary Communication/) OR ((collaborat*.ti,ab. OR Coordinat*.ti,ab. OR Integrat*.ti,ab. OR Teamwork.ti,ab.) AND (disciplin*.ti,ab. OR profession*.ti,ab. OR institution*.ti,ab. OR organisation*.ti,ab. OR occupation*.ti,ab. OR agenc*.ti,ab. OR sector*.ti,ab. OR care.ti,ab. OR team.ti,ab.))

AND

(General Practice.ti,ab. OR Community Clinic.ti,ab. OR Community Practice.ti,ab. OR Community Health.ti,ab. OR Community setting.ti,ab. OR Community-based Care.ti,ab. OR Primary Health.ti,ab. OR Primary Care.ti,ab. OR Primary-care.ti,ab. OR Private Practice.ti,ab. OR Family Practice.ti,ab. OR
Ambulatory Practice.ti,ab. OR Home care.ti,ab. OR General Practice/ OR Family Practice/ OR
Primary Health Care/ OR Community Health Services/ OR Ambulatory Care/ OR Chronic care model.ti,ab. OR medical home.ti,ab. OR healthcare home.ti,ab.)

AND

((patient*.ti,ab. OR Consumer*.ti,ab. OR Client*.ti,ab.)

ADJ3

(Experience*.ti,ab. OR Perception*.ti,ab. OR Perceive*.ti,ab. OR Perspective*.ti,ab. OR View*.ti,ab.))
