# Supplementary material for: What do patients experience? Interprofessional collaborative practice for chronic conditions in primary care: an integrative review
Source: BMC Prim Care. 2022 Jan 14;23:8. doi: 10.1186/s12875-021-01595-6 (PMC8759162; doi:10.1186/s12875-021-01595-6)
Supplement: Supplementary file 3 — Additional file 3. Description of Included Studies. [file 12875_2021_1595_MOESM3_ESM.docx]

| Author (year)  Country | Design & Primary Care Setting | Patient participants (n = P)  Chronic Condition(s) | Aim related to review question (patient experience) | Intervention/Exposure (***I***)  + Comparator/Control (***C***) | Themes and sub-themes related to study |
| --- | --- | --- | --- | --- | --- |
| Abdulrhim  (2021)  Qatar | Qualitative –  Interviews  Primary Healthcare Clinic | Patients (*n*=12)  T2DM | To explore the value of collaborative care model in diabetes care at a primary healthcare setting from the perspectives of patients with diabetes | ***I:*** Collaborative Care Model provides personalised patient education and treatment plans by a MDT of physicians, nurses and pharmacists.  ***C:*** N/A | Interacting with healthcare teams:   - widening the patient network, - connecting with professionals - looking beyond the condition - overcoming chronic condition collectively   Valuing Convenient Healthcare:   - sharing space & time - planning care creates structure - affording care |
| Balasubramanian (2017)  USA | Mixed Methods (Qualitative section – Interviews)  Primary Care & Community Mental Health Centres | Patients (*n*=25)  Major Depression | To evaluate effect of integrated care, adapted to local context, on depression severity and patients’ experience of care. | ***I:*** Integration of behavioural and primary health care across 5 practices – each with different integrated care approaches. All included: primary care and behavioural health professionals, screening for behavioural/mental health conditions, provided behavioural health counselling.  Part of Advancing Care Together (ACT) program.  ***C:*** N/A | Interacting with healthcare teams:   - widening the patient network, - connecting with professionals - looking beyond the condition - overcoming chronic condition collectively   Valuing Convenient Healthcare:   - sharing space & time - coordinating care - valuing the general practitioner role |
| Banfield (2017)  Australia | Qualitative – Interviews  (pilot study)  Super Clinic | Consumers (*n*=19) Had a minimum of one chronic condition. Conditions not described. | To explore consumers’ and providers’ concepts, expectations and experience of integrated care. | ***I:*** GP Super clinic – co-location and integration of general practice with allied health (AH) and other services providers to support those with chronic disease through patient-centred care and effective use of information technology. Team: 12 GPs, 6 practice nurses, 11 admin staff and 9 AH.  ***C:*** N/A | Interacting with healthcare teams:   - widening the patient network, - connecting with professionals - looking beyond the condition - overcoming chronic condition collectively   Valuing Convenient Healthcare:   - sharing space & time - planning care creates structure - coordinating care - valuing the general practitioner role - affording care |
| Banfield (2019) Australia | Quantitative – Descriptive – Survey  General Practice | Patients (*n*=18)  T2DM and Mental Health Conditions | To examine consumer experiences of GP care plans for mental disorders and Type II diabetes. | ***I***: General practice care plans (Chronic Disease Management (CDM) plan and Mental Health Care Plan (MHCP)). CDM enable GPs to plan and coordinate the health care of people with chronic medical conditions through collaborative preparation and review of plans with consumers. MHCP designed to encourage collaborative care between GPs, consumers, and other services providers and to provide access to psychiatrists, psychologists, and other professionals.  ***C:*** N/A | Interacting with healthcare teams:   - widening the patient network, - overcoming chronic condition collectively   Valuing Convenient Healthcare:   - planning care creates structure - coordinating care - valuing the general practitioner role - affording care |
| Burridge (2016) Australia | Qualitative – Interviews   General Practice Diabetes Services | Patients (*n*=30)  T2DM | To investigate patients’ perceptions and experiences of T2DM, self-care and engagement with GP-led integrated diabetes care | ***I***: GP-led model: care is provided by a co-located MDT (an endocrinologist, advanced-skilled GPs, diabetes educator and a podiatrist). Other AH was referred to based on patient needs.  ***C:*** N/A | Interacting with healthcare teams:   - widening the patient network, - connecting with professionals - looking beyond the condition - overcoming chronic condition collectively   Valuing Convenient Healthcare:   - planning care creates structure - coordinating care - valuing the general practitioner role   Engaging Self-care:   - engaging passively is circumstantial - engaging actively and leading care |
| Burridge (2017)  Australia  12-month follow-up study of Burridge 2016 | Qualitative – Interviews   General Practice Diabetes Services | Patients (*n*=25)  T2DM | To explore patients’ view of a new model of integrated care for patients with type 2 diabetes. | ***I***: GP-led model: care is provided by a co-located MDT (an endocrinologist, advanced-skilled GPs, diabetes educator and a podiatrist). Other AH was referred to based on patient needs.  ***C:*** N/A | Interacting with healthcare teams:   - widening the patient network, - connecting with professionals - looking beyond the condition - overcoming chronic condition collectively   Valuing Convenient Healthcare:   - sharing space & time - coordinating care - valuing the general practitioner role   Engaging Self-care:   - engaging passively is circumstantial - engaging actively and leading care |
| Butters (1993)  UK | Mixed Methods – Interviews and Questionnaires  Community PCMH | Patients (*n*=19) and informal carers (*n*=8)  HIV/AIDS | To compare the views of palliative care reports by patients, informal carers, and the Community Care Team (CCT) | ***I***: CCT is a MDT consisting of 2 doctors (a consultant and registrar), 3 clinical nurse specialists, a dietitian, an occupational therapist, and administrator and a research assistant aimed at supporting HIV/AIDs patients and their carers.  ***C:*** N/A | Interacting with healthcare teams:   - widening the patient network, - connecting with professionals   Valuing Convenient Healthcare:   - sharing space & time - affording care   Engaging Self-care:   - engaging passively is circumstantial |
| Davis (2018)  USA | Qualitative – Interviews  Primary care and Community Mental Health Centres (CMHC) | Patients (*n*=24)  Mental health conditions | To examine patients’ experiences of care in community-based settings integrative behavioural health and primary care. | ***I***: Advancing Care Together (ACT) program (4-year program). 5 practices – with varying activities to deliver integrated care (behavioural and primary care) Private, not-for-profit CMHC (n=2); Clinician-owned primary care clinic (n=2); Private, not-for-profit FQHC (n=1). Strategies include co-location of professionals, systematic screening of pts, transitions of care to appropriate services.  ***C:*** N/A | Interacting with healthcare teams:   - widening the patient network, - connecting with professionals - looking beyond the condition - overcoming chronic condition collectively   Valuing Convenient Healthcare:   - sharing space & time - planning care creates structure - affording care |
| Drainoni (2014)  USA | Mixed-Methods  Survey and Focus Groups  Integrated Primary Care Clinic | Patients (*n*=212 for survey, *n*=40 focus groups)  HIV and substance use disorders | To assess patients’ views of [a team-based model of integrated care], evaluate key elements of success and provide recommendations for other programs | ***I***: A team-based model of integrated care within the primary care setting for HIV-infected substance users and substance users at risk for contracting HIV. The Facilitated Access to Substance abuse Treatment with Prevention And Treatment of HIV (FAST PATH) program consisted of a comprehensive MDT assessment, diagnosis, and treatment plan developed by a team (a physician, nurse and an addiction counsellor case manager).  ***C:*** N/A | Interacting with healthcare teams:   - widening the patient network, - connecting with professionals - looking beyond the condition - overcoming chronic condition collectively   Valuing Convenient Healthcare:   - sharing space & time - coordinating care - valuing the general practitioner role - affording care |
| Ede (2015)  USA | Quantitative Descriptive – Survey  Integrated primary healthcare practices | Patients (*n* = 51)  Mental and behavioural health conditions | To describe and examine perceptions and level of satisfaction with integrated care. | ***I***: Integrated care involved behavioural health working within and as part of primary care. 5 practices. Model of care: co-location – mental health is integrated into physical health (n=3); reverse co-location – physical health is integrated into mental health (n=1); collaborative care – two-sites collaborating (n=1).  ***C:*** N/A | Interacting with healthcare teams:   - widening the patient network, - connecting with professionals - looking beyond the condition   Valuing Convenient Healthcare:   - sharing space & time - planning care creates structure   Engaging Self-care:   - engaging actively and leading care |
| Foster (2015) Australia | Qualitative – Interviews  General Practice | Patients (*n*=23)  Asthma, chronic respiratory disease, diabetes and heart problems. | To explore the views of primary care patients who were currently in receipt of Medicare-funded team care for management of chronic disease | ***I***: Participants received a CDM, a team care arrangement which included GPs and individual AH services – up to 5 government rebated sessions per year with AH (e.g. physiotherapist, dietetics, podiatry, etc).  ***C:*** N/A | Interacting with healthcare teams:   - widening the patient network, - connecting with professionals   Valuing Convenient Healthcare:   - sharing space & time - planning care creates structure - coordinating care - valuing the general practitioner role - affording care   Engaging Self-care:   - engaging passively is circumstantial - engaging actively and leading care |
| Freyens (2005)  Belgium | Mixed-Methods  Ambulatory Care | Patients (*n*=25)  Diabetes | To implement an ambulatory multidisciplinary programme and evaluate feasibility of an educational module in type 2 diabetic patients followed in primary care. | ***I***: A patient orientated MDT intervention: 4 GPs collaborated with an integrated health medical centre consisting of: 2 dietitians, 4 psychologist and 4 nurses all working together and cooperating on the same groups, while maintaining an independent status, care included an MDT meeting half-way where each participant was discussed. Average intervention = 9 appointments over 3-4months.  ***C:*** N/A | Interacting with healthcare teams:   - widening the patient network, - connecting with professionals - looking beyond the condition   Valuing Convenient Healthcare:   - valuing the general practitioner role - affording care   Engaging Self-care:   - engaging actively and leading care |
| Fu (2018)  UK | Qualitative – Interviews  Public chronic pain clinic | Patients (*n*=22)  Chronic pain | To explore the nature of patient-professional partnerships and its related factors that create facilitators and barriers to patients’ self-management ability. | ***I***: Health professionals (pain nurses, physiotherapists, health care trainers and doctors) worked together to ensure patients received treatment and care that were tailored to their individual needs.  ***C:*** N/A | Interacting with healthcare teams:   - widening the patient network - connecting with professionals - looking beyond the condition - overcoming chronic condition collectively   Valuing Convenient Healthcare:   - sharing space & time - planning care creates structure - coordinating care - valuing the general practitioner role   Engaging Self-care:   - engaging actively and leading care |
| Gorina (2014)  Spain | Quantitative – non-randomized study – cross-sectional  Home Care | Patients (Integrated Model *n* = 67, Dispensed Model *n*= 91) Chronic illness, degenerative illness or multi-organ disorders. | To compare the degree of satisfaction of users who receive home care through the IM against those who do so through the DM | ***I***: Dispensed Model (DM) – interdisciplinary teams – medical, nursing, and social work professionals offer holistic care to patients and their families dedicated to home care for all their practice hours.  ***C:***  Integrated Model (IM) – interdisciplinary teams – medical, nursing and social work professionals offer holistic care to patients and their familied dedicated to home care for 1 hour of the day. | Interacting with healthcare teams:   - widening the patient network - connecting with professionals - overcoming chronic condition collectively   Valuing Convenient Healthcare:   - sharing space & time   Engaging Self-care:   - engaging actively and leading care |
| Grimmer-Somers (2010)  Australia | Mixed Methods  (Qualitative – interviews relate to review)  General Practice | Patients (*n* = 59)  T2DM | To describe the patient perspective of EPC, and what they felt they had gained from participation in it. | ***I***: Under the Enhanced Primary Care (EPC) program, AH (diabetes educators, dietitians and podiatrists) provided services within or close to GPs’ rooms. EPC initiative enabled up to 5 sessions per year with AH – pilot program ran for 8 months.  ***C:*** N/A | Interacting with healthcare teams:   - widening the patient network, - connecting with professionals - looking beyond the condition   Valuing Convenient Healthcare:   - sharing space & time - coordinating care - valuing the general practitioner role - affording care   Engaging Self-care:   - engaging passively is circumstantial - engaging actively and leading care |
| Grohmann (2017)  Canada | Qualitative – interviews  Family Practice | Patients (*n*=23) T2DM | To explore patients' perspectives on care received from diabetes education teams (a registered nurse and a registered dietitian) integrated into primary care. | ***I***: Diabetes education teams (a registered nurse and registered dietitian) were integrated into primary care practices (primary care physicians). Patients received half an hour appointment with the registered nurse and registered dietitian or with both.  ***C:*** N/A | Interacting with healthcare teams:   - widening the patient network, - connecting with professionals - overcoming chronic condition collectively   Valuing Convenient Healthcare:   - sharing space & time - coordinating care - valuing the general practitioner role   Engaging Self-care:   - engaging actively and leading care |
| Grol  (2020)  The Netherlands | Mixed Methods – Interviews and Survey for a Social Network Analysis  General Practice | Patients (*n*=44)  Frail older adults with ≥2 chronic conditions | To gain more insight into the experiences of frail older patients with integrated multidisciplinary care by mapping the care networks of this patient group and their perception of the interconnection between professional and informal caregivers | ***I***: Patients are registered with a GP. GP provides comprehensive and patient-oriented approach with high continuity and coordination of care.  4 GP practices were chosen – with heterogeneous characteristics (location and population serviced, years experience with MDC and scale of GP setting.  ***C:*** N/A | Interacting with healthcare teams:   - widening the patient network,   Valuing Convenient Healthcare:   - coordinating care - valuing the general practitioner role   Engaging Self-care:   - engaging passively is circumstantial - engaging actively and leading care |
| Hannane (2017)  France | Qualitative – interviews  General Practice | Patients (*n*= 30)  Asthma | To explore the perceptions of French adult asthma patients on their care pathway. | ***I***: French collaborative primary care – GPs are assigned the role of care coordinator with a nurse involved in monitoring of chronic conditions.  ***C:*** N/A | Interacting with healthcare teams:   - widening the patient network, - connecting with professionals - looking beyond the condition - overcoming chronic condition collectively   Valuing Convenient Healthcare:   - sharing space & time - planning care creates structure - coordinating care - valuing the general practitioner role   Engaging Self-care:   - engaging passively is circumstantial |
| Hepworth (2013)  Australia | Qualitative – Interviews  General Practice – Integrated Primary Care | Patients (*n*=10)  T2DM | To explore how a new model of integrated primary/secondary care for type 2 diabetes management related to improved diabetes management in a selected group of patients. | ***I***: Brisbane South Complex Diabetes Service – delivered in a large general practice. A multiprofessional team – an endocrinologist, advanced-skilled GPs, a credential diabetes educator and a podiatrist provided care.  ***C:*** N/A | Interacting with healthcare teams:   - widening the patient network, - connecting with professionals - looking beyond the condition - overcoming chronic condition collectively   Valuing Convenient Healthcare:   - planning care creates structure - coordinating care - valuing the general practitioner role   Engaging Self-care:   - engaging passively is circumstantial - engaging actively and leading care |
| Hudon (2016) Canada | Qualitative – Interviews and focus groups  Family Practice | Patients (*n*=25)  Chronic Disease – diabetes, CVD, respiratory disease, musculoskeletal disease or chronic pain. | To examine the experience of patients and their family members with care integration as part of a primary care case management (CM) intervention. | ***I***: A 6-month CM intervention by nurses in 4 family medicine groups – primary care physicians are grouped together to collaborate with nurses to offer primary care services. CMs were registered nurses who evaluated, planned, implemented, coordinated and prioritised options and services according to patient needs in close collaboration with involved partners.  ***C:*** N/A | Interacting with healthcare teams:   - widening the patient network, - connecting with professionals - overcoming chronic condition collectively   Valuing Convenient Healthcare:   - planning care creates structure - coordinating care - valuing the general practitioner role - affording care   Engaging Self-care:   - engaging passively is circumstantial |
| Jones (2011)  Australia | Qualitative – Interviews  General Practice | Patients (*n*=10)  T2DM | To explore both sets of uses’ perceptions and experiences of using CDMS during the interventions phase of the project. | ***I***: Electronic care planning system – supports the management of chronic disease and collaboration among the care team and the person with the condition. An electronic General practice Management Plan – helps GPs manage patients who require interdisciplinary care – involves a GP and at least two other health professionals.  ***C:*** N/A | Interacting with healthcare teams:   - widening the patient network, - connecting with professionals - overcoming chronic condition collectively   Valuing Convenient Healthcare:   - planning care creates structure - coordinating care - valuing the general practitioner role   Engaging Self-care:   - engaging passively is circumstantial - engaging actively and leading care |
| Karlsson (2015)  European countries: England, Estonia, Finland, France, Germany, Netherlands, Spain and Sweden | Qualitative- Focus Groups  Multiple primary care settings - home care, self-help groups, counselling services and memory or dementia associations | Patients and informal caregivers (*n*=137)  Dementia | To investigate persons with dementia and their informal caregivers’ views of inter-sectoral information, communication and collaboration throughout the trajectory of care, from diagnosis to end-of-life care in eight European countries. | ***I***: No Intervention. Trajectory of dementia care in 8 European countries (England, Estonia, Finland, France, Germany, The Netherlands, Spain and Sweden).  ***C:*** N/A | Interacting with healthcare teams:   - widening the patient network, - connecting with professionals - overcoming chronic condition collectively   Valuing Convenient Healthcare:   - sharing space & time - coordinating care - valuing the general practitioner role   Engaging Self-care:   - engaging actively and leading care |
| Klarare (2017)  Sweden | Qualitative – Interviews  Home Care | Patients (*n*=6)  Family members (*n*=7)  Malignant Cancer | To investigate how the team’s work is manifested in care episodes narrated by patients and families in specialized palliative home care (SPHC) | ***I***: 4 SPHC – comprised of 30 health professionals – including physicians, registered nurses, social workers, physical therapists, and occupational therapists. Team leaders were physicians or nurses. Teams were responsible for: symptom management, treatment and nutritional support, social and existential issues. Teams were organised so a physician is paired with a group of nurses – where a patient is designated a nurse responsible for care planning.  ***C:*** N/A | Interacting with healthcare teams:   - widening the patient network, - connecting with professionals - looking beyond the condition - overcoming chronic condition collectively   Engaging Self-care:   - engaging actively and leading care |
| Knowles (2015)  UK | Qualitative – Interviews  General Practice | Patients (*n*=29)  Depression and either CHD or Diabetes | To examine how patients understood and experiences the integration of mental and physical health care | ***I***: Part of the COINCIDE pragmatic trial of collaborative care – integrating low-intensity psychological intervention with the context of routine primary care management of depression. Psychological therapists act as case managers, working with practice nurses to enhance the integration of mental and physical healthcare through improved interprofessional communication. 8 sessions over 12 weeks. GPs were involved in the team triad.  ***C:*** N/A | Interacting with healthcare teams:   - widening the patient network, - connecting with professionals - looking beyond the condition - overcoming chronic condition collectively   Valuing Convenient Healthcare:   - sharing space & time |
| MacPhail (2009)  USA | Qualitative – Interviews  Primary Medical Centre | Patients (*n*=65)  Diabetes | To identify processes by which providers worked together to provide care using an electronic health record (EHR) and to examine factors supporting coordination of care. | ***I***: Adult diabetes MDTs in 4 diverse models of care within an integrated health system where all providers use a comprehensive shared EHR.  ***C:*** N/A | Interacting with healthcare teams:   - widening the patient network, - connecting with professionals - overcoming chronic condition collectively   Valuing Convenient Healthcare:   - sharing space & time - planning care creates structure - coordinating care - valuing the general practitioner role |
| Maeng (2013)  USA | Quantitative Descriptive – Survey  PCMH | Patients (*n*=499 – intervention, *n*=356 – comparison)  Asthma, CAD, cancer, CHF, CKD, COPD, depression, diabetes, ESRD, hypertension. | To evaluate the impact of Proven Health Navigator (PHN) on patient experience of care. | ***I***: PHN – blended aspects of the chronic care and patient-centred primary care models.  An alliance between insurance company, patients and their families, primary care physicians, and other health care partners.  Core components of model: patient-centred primary care, population management, medical neighbourhood, quality outcomes and value-based reimbursement model.  ***C:***  Non-PHN | Interacting with healthcare teams:   - widening the patient network, - connecting with professionals   Valuing Convenient Healthcare:   - sharing space & time - coordinating care   Engaging Self-care:   - engaging passively is circumstantial |
| McDonald (2012)  Australia | Qualitative – Interviews  Multiple primary care settings: Two GP practices running diabetes clinics and a diabetes centre. | Patients (*n*=8)  Diabetes | To explore the influence of power dynamics and trust on collaboration between health professionals involved in the management of diabetes and their impact on patient experience. | ***I***: Primary and community-based health services for adults with T2DM. General practices running diabetes clinics and a diabetes centre. HPs in these services include dietitian, GP, practice nurse, medical specialist, optometrist, pharmacists, physiotherapist, podiatrist, Aboriginal health education office, community nurse, diabetes educator, fitness instructor and Aboriginal community worker – across private and public health services  ***C:*** N/A | Interacting with healthcare teams:   - widening the patient network, - connecting with professionals - overcoming chronic condition collectively   Valuing Convenient Healthcare:   - planning care creates structure - coordinating care - valuing the general practitioner role   Engaging Self-care:   - engaging passively is circumstantial - engaging actively and leading care |
| Miller-Rosales  (2020)  USA | Cluster-randomized trial (Quantitative Descriptive section) – survey  Community Health Centres | Patients (*n*= 1277)  T2DM | To examine whether care team role expertise is associated with patients' experience of chronic care and whether the relationship is stronger for small community health centre (CHC) cites | ***I:*** CHC cites with a range of professionals, including community health workers, diabetes educators, nutritionists, pharmacists, mental health providers and other general staff, as well as the physician.  ***C:*** A small (<250 patients) vs large (>250 patients) CHC site. | Interacting with healthcare teams:   - widening the patient network,   Valuing Convenient Healthcare:   - sharing space & time |
| Otero-Sabogal  (2010)  USA | Mixed Methods (Quantitative Descriptive section) – Telephone Survey  Primary Health Clinic | Patients (*n*=31)  T2DM | To improve self-management among patients with type 2 diabetes incorporating community health workers (CHW) as members of a clinical team. | ***I***: Team-based self-management intervention including an extended role of the CHW in a MDT clinical team working with primary care physician and licensed practitioners.  CHW main roles involve patient outreach and education whilst working within a team. Programme included two primary care physicians, three CHWs, a diabetes educator and a social worker.  ***C:*** N/A | Interacting with healthcare teams:   - widening the patient network, - connecting with professionals - looking beyond the condition - overcoming chronic condition collectively   Valuing Convenient Healthcare:   - sharing space & time - planning care creates structure   Engaging Self-care:   - engaging actively and leading care |
| Penney  (2016)  USA | Qualitative – Focus groups and Interviews  Primary Care | Patients (*n*=90) Chronic musculoskeletal pain (CMP) | To describe the communication system surrounding the management of chronic pain from the perspectives of CMP patients. | ***I***: Acupuncture and chiropractic implemented in usual care of CMP with a Complementary Health Plan (patients are referred by their primary care physician or a specialist to receive a limited number of visits with an acupuncturist or chiropractor).  ***C:*** N/A | Interacting with healthcare teams:   - widening the patient network, - connecting with professionals - looking beyond the condition - overcoming chronic condition collectively   Valuing Convenient Healthcare:   - coordinating care - valuing the general practitioner role - affording care   Engaging Self-care:   - engaging actively and leading care |
| Pullon  (2011)  New Zealand | Qualitative – Interviews  General Practice | Patients (*n*=4)  Asbestosis, COPD, Depression, Neurofibromatosis, T2DM, Tuberculosis | To test both the feasibility of undertaking a collaborative method of enquiry as a means of investigating patient perceptions about teamwork in the context of their current health care | ***I***: CarePlus Programme – encourages goal setting by patients and self-management. Teamwork of usual health professionals in NZ suburban medium-sized general practice settings with GPs and practice nurses.  ***C:*** N/A | Interacting with healthcare teams:   - widening the patient network, - connecting with professionals - overcoming chronic condition collectively   Valuing Convenient Healthcare:   - sharing space & time - coordinating care - valuing the general practitioner role - affording care   Engaging Self-care:   - engaging passively is circumstantial - engaging actively and leading care |
| Purcell  (2019)  USA | Qualitative – Interviews  Primary Care – VA medical centers (outpatient campuses) | Veterans (*n*=41)  Chronic pain and opioid misuse | To examine the patient experience with a pain care model: interdisciplinary, integrated pain teams (IPTs) embedded in primary care | ***I***: IPT provides integrate, interdisciplinary chronic pan care within VA primary care settings. Patients see a medical provider, psychologist and a pharmacist as a unified interdisciplinary team. All professionals are present for the initial appointment and appropriate follow-ups booked. Personalized care plans are created with team and patient. Other services such a physiotherapy and acupuncture are referred outside of the team.  ***C:*** N/A | Interacting with healthcare teams:   - widening the patient network, - connecting with professionals - looking beyond the condition - overcoming chronic condition collectively   Valuing Convenient Healthcare:   - sharing space & time - planning care creates structure - coordinating care - valuing the general practitioner role   Engaging Self-care:   - engaging passively is circumstantial - engaging actively and leading care |
| Quigley  (2021)  Australia | Qualitative – Interviews  Primary Care – General Practice | Patients (*n*=17) and informal carers (*n*=9)  Chronic conditions – not specified | To explore the experiences of older people and their carers of a unique model of integrated care that provides specialist geriatric intervention in primary care for older adults with complex needs. | ***I:*** Model for older adults with complex needs. Assigned an enablement officer (nursing or AH professional), general practitioner and geriatrician. Shared medical records, and case conferencing part of model.  ***C:*** N/A | Interacting with healthcare teams:   - widening the patient network, - connecting with professionals - looking beyond the condition - overcoming chronic condition collectively   Valuing Convenient Healthcare:   - sharing space & time - planning care creates structure - coordinating care - valuing the general practitioner role - affording care   Engaging Self-care:   - engaging actively and leading care |
| Reiss-Brennan  (2014)  USA | Quantitative Descriptive  Primary Care | Patients (*n*=59; across three clinics with different levels of IPCP)  Depression | To evaluate the impact of mental health integration on depression care | ***I***: Mental Health Integration (MHI) – team-based approach incorporating mental health including the patient and family and are operationalized at the clinic improving both physician and staff communication. Team: PCPs, support staff, practice managers, mental health professionals, community advocates, patient, and family.  Adoption Clinic – adopted MHI for >2 years.  Routinized Clinic – adopted MHI for >6 years.  ***C:***  Potential clinics – are trained in the MHI team CPM but have not yet adopted MHI plan. | Interacting with healthcare teams:   - widening the patient network, - connecting with professionals - looking beyond the condition - overcoming chronic condition collectively   Valuing Convenient Healthcare:   - sharing space & time - coordinating care - valuing the general practitioner role |
| Roberge  (2016)  Canada | Qualitative – Interviews  Family Medical Practice | Patients (*n*=10)  Anxiety and/or depression and a multimorbid physical condition | To explore the perceived needs, barrier and facilitator for the delivery of mental health care for patients with coexisting common mental disorders and chronic diseases in primary care from the clinician and patient perspectives. | ***I***: 3 primary care clinics with family physicians, nurses and nurse practitioners, psychologists and social workers, pharmacists, clinical coordinators, medical residents, and consultant psychiatrists.  ***C:*** N/A | Interacting with healthcare teams:   - widening the patient network, - connecting with professionals - looking beyond the condition - overcoming chronic condition collectively   Valuing Convenient Healthcare:   - coordinating care - valuing the general practitioner role - affording care |
| Ryan, 2020  UK | Qualitative - Interviews  National Health Service (NHS) Primary Care Trust | Patients (*n*=14)  Sciatica | To explore how people experience being managed for sciatica within an NHS pathway. | ***I***: Outpatient physiotherapist service in an NHS community trust where the person’s GP referred them to the service. MDT included GP, physio, specialist physio at least, with some individuals having a spinal specialist, chiropractor, and/or podiatrist.  ***C:*** N/A | Interacting with healthcare teams:   - widening the patient network, - connecting with professionals - looking beyond the condition - overcoming chronic condition collectively   Valuing Convenient Healthcare:   - sharing space & time - planning care creates structure - coordinating care - valuing the general practitioner role - affording care   Engaging Self-care:   - engaging passively is circumstantial - engaging actively and leading care |
| Ryrie  (2001)  UK | Qualitative – Interviews  Community Rehabilitation Program | Patients (*n*=15)  Mental Health Conditions | To describe the client perceptions of the service and community rehabilitation team (CRT). | ***I***: CRT consisted of a psychologist (team leader), OT, mental health nurse, vocational rehab officer, senior support worker and psychology assistant. Team is responsible to provide case management including the development a rehabilitation remit for individuals including daily living skills, social integration, vocational, occupational or education needs, and psychological interventions.  ***C:*** N/A | Interacting with healthcare teams:   - connecting with professionals - looking beyond the condition - overcoming chronic condition collectively   Valuing Convenient Healthcare:   - planning care creates structure   Engaging Self-care:   - engaging passively is circumstantial - engaging actively and leading care |
| Simpson (2008)  UK | Qualitative – Interviews  General Practice | Patients (*n*= 13)  Depression | To explore the experiences of patients who receive treatment for depression during a ‘Phase II’ platform trial of collaborative care in the UK. | ***I***: Collaborative Care included four key elements:  Multiprofessional approach to patient care (Case manager (OT, nurse, counsellor), GP, specialist mental health medical and psychological therapies clinicians); Structured management plan (medication and behavioural support plan)  Scheduled patient follow-ups; Enhanced interprofessional communication (regular patient-specific written feedback to GPs via electronic records).  ***C:*** N/A | Interacting with healthcare teams:   - widening the patient network, - connecting with professionals - overcoming chronic condition collectively   Valuing Convenient Healthcare:   - coordinating care - valuing the general practitioner role |
| Soderberg (2015)  Sweden | Qualitative – Interviews  Home Care | Patients (*n*=8)  Chronic Heart Failure | To describe experience of security and insecurity in patients with chronic heart failure who were treated by Advanced Home Care (AHC) in a city in Sweden. | ***I***: Advanced Home Care: Multi-professional team that plans and works to reduce the deterioration of illness.  ***C:*** N/A | Interacting with healthcare teams:   - widening the patient network, - connecting with professionals - looking beyond the condition   Valuing Convenient Healthcare:   - sharing space & time   Engaging Self-care:   - engaging passively is circumstantial |
| Sorensen  (2020)  Norway | Qualitative – Interviews  General Practice | Patients (*n*=11)  T1DM and T2DM | To explore how patients with diabetes and multimorbidity experience self-management supported by general practitioners (GPs) nurses and medical secretaries in Norwegian general practice | ***I:*** A diabetes specialist nurse, nurse or medical secretary worked with GPs to provide patient-centred care.  ***C:*** N/A | Interacting with healthcare teams:   - widening the patient network, - connecting with professionals - looking beyond the condition - overcoming chronic condition collectively   Valuing Convenient Healthcare:   - sharing space & time - coordinating care - valuing the general practitioner role   Engaging Self-care:   - engaging passively is circumstantial - engaging actively and leading care |
| Sundstrom  (2017)  Sweden | Qualitative – Interviews  Home Care | Patients (*n*=10)  Multiple Conditions | To gain a deeper understanding of the Health and Social Care Planning in Collaboration (HSCPC)-meeting from the perspectives of older persons, family members, and professionals | ***I***: 2 municipals participated in the HSCPC and worked out its own strategy, including who should participate, coordinate and facilitate the meeting, how the meeting should be conducted, what issues should be discussed and where the meeting would be held. Team: GP, social worker, home care aid, community nurse, occupational- or physiotherapist and family.  ***C:*** N/A | Interacting with healthcare teams:   - connecting with professionals   Valuing Convenient Healthcare:   - sharing space & time - planning care creates structure - coordinating care - valuing the general practitioner role   Engaging Self-care:   - engaging passively is circumstantial |
| Talabani  (2017)  Sweden | Qualitative – Interviews  Home Care | Patients (*n*=12)  Heart Failure | To describe patients’ experiences of a new model of person-centred integrated HF and palliative care at home. | ***I***: The Palliative advanced home caRE and heart failurE caRe (PREFER) study – is a multidisciplinary approach to integrating palliative advanced home care and HF clinical care. Team included: HR nurses, palliative care nurses, palliative care physician, cardiologist, physiotherapist and occupational therapist.  Key components were: specialised care, team-based care, total care, home-cased care, key individual, PCC, structured care, easy access, support to close relatives and support to team members.  ***C:*** N/A | Interacting with healthcare teams:   - widening the patient network, - connecting with professionals - looking beyond the condition - overcoming chronic condition collectively   Valuing Convenient Healthcare:   - sharing space & time   Engaging Self-care:   - engaging passively is circumstantial |
| Tan  (2013)  Australia | Qualitative – Interviews  General Practice | Patients (*n*=18)  Asthma, depression, diabetes, hypertension and osteoporosis. | To explore general practice staff, pharmacist and patient experiences with pharmacist services in Australian general practice clinic within the Pharmacists in Practice Study (PIPS) | ***I***: 2 general practices (1 private practice and 1 community health centre) where PIPS pharmacists are integrated for 6-months and collaborate with the GP to provide extensive pharmaceutical and medical care. Pharmacists conducted consultations with clients referred by GPs, had access to medical records and discussed patient issues with GP.  ***C:*** N/A | Interacting with healthcare teams:   - connecting with professionals   Valuing Convenient Healthcare:   - sharing space & time - coordinating care - valuing the general practitioner role |
| Taylor  (2018)  UK | Qualitative – Interviews  General Practice | Patients (*n*=18)  Depression | To explore patients’ and professionals’ views on collaborative care developed for older people, and how this model could be implemented at scale | ***I***: Low-intensity intervention of collaborative care delivered by a case manager for an average of 6 sessions over 7-8 weeks alongside usual GP care. 5 components of collaborative care: patient centred assessment, symptom monitoring, medication management, active follow-up and behavioural activation.  ***C:*** N/A. Control group was not interviewed. | Interacting with healthcare teams:   - widening the patient network - connecting with professionals   Valuing Convenient Healthcare:   - planning care creates structure - coordinating care - valuing the general practitioner role |
| Tiozzo  (2019)  Italy | Quantitative non-randomised studies – Cohort  General Practice | Patients (*n*=68) from intervention group Chronic heart failure and multimorbidity | To describe the impact of a care management program (CMP) developed in the Veneto region for patients affected by chronic heart failure and multimorbidity. | ***I***: “Exposed” group - CMP was provided by a team: specially trained care manager nurse and a GP. Worked closely to provide patient-centred and coordinated care including baseline assessment, care planning and proactive follow-up.  ***C:*** “Unexposed” group – continued to receive usual care from their established GP. | Interacting with healthcare teams:   - looking beyond the condition - overcoming chronic condition collectively   Valuing Convenient Healthcare:   - planning care creates structure   Engaging Self-care:   - engaging actively and leading care |
| van Dongen (2017)  The Netherlands | Qualitative – Focus Groups  Primary Care | Patients (*n*=7)  Asthma, blind since childbirth, breast cancer, cardiovascular, multiple sclerosis, osteoarthritis, spinal cord injury. | To explore the patient’s perspectives regarding Interprofessional Team (IPT) meetings in primary care. | ***I***: Participants had to be exposed to interprofessional collaboration and meetings. Interprofessional teams include family physicians, practice nurses, occupational therapists, district nurses and pharmacists. Main objective of meetings was to discuss patient care plans.  ***C:*** N/A | Interacting with healthcare teams:   - widening the patient network, - connecting with professionals - overcoming chronic condition collectively   Valuing Convenient Healthcare:   - sharing space & time - planning care creates structure   Engaging Self-care:   - engaging passively is circumstantial - engaging actively and leading care |
| Walker  (2013)  USA | Qualitative – Focus Groups  Primary Care Clinics | Patients (*n*=44)  Diabetes, hypertension, chronic lung disease, depression, chronic kidney disease, osteoarthritis, congestive heart failure, mild cognitive impairment) | To explore the patient experience with the functional domains of integrated care | ***I***: Integrated care: “patient care that is coordinated across professionals, facilities, and support systems; continue over time and between visits; tailored to the patients’ needs and preferences; and based on shared responsibility between patient and caregivers for optimizing health”  ***C:*** N/A | Interacting with healthcare teams:   - widening the patient network, - connecting with professionals - overcoming chronic condition collectively   Valuing Convenient Healthcare:   - planning care creates structure   Engaging Self-care:   - engaging actively and leading care |
| Wilson  (2019)  USA | Qualitative – Interviews  Primary Care Clinics | Patients (*n*=48)  Multimorbidity (≥2 chronic conditions). | To understand patients’ perceptions of the consent process, their reasons for choosing to participant, and their experiences receiving chronic care management (CCM) services | ***I***: CCM – available to Medicare beneficiaries with 2 or more chronic conditions. Providers are to enhanced patients’ access to continuous and coordinated care, including care management. Providers include physicians, nurse practitioners and physician assistants, clinical nurse specialists, and nurse midwives. CCM services include developing a care plan in collaboration with the patient, communicating with other health professionals and services, 24/7 access to care for urgent needs and ongoing medication management. Electronic records are used and shared.  ***C:*** N/A | Interacting with healthcare teams:   - connecting with professionals   Valuing Convenient Healthcare:   - sharing space & time - planning care creates structure - coordinating care - valuing the general practitioner role - affording care |

ADLs, activities of daily living; AH, Allied Health; AIDS, autoimmune disease syndrome; CAD, coronary artery disease; CHD, coronary heart disease; CHF, congestive heart failure; CHW, Community Health Workers; CKD, chronic kidney disease; COPD, Chronic obstructive pulmonary disease; EPC, Enhanced Primary Care; ESRD, end-stage renal disease; GP, General Practitioner; HIV, Human Immunodeficiency Virus; MDT, multidisciplinary team; PCMH, patient-centred medical home; PCP, Primary Care Practitioner/Provider; T2DM, Type 2 Diabetes Mellitus.
