# Supplementary material for: What do patients experience? Interprofessional collaborative practice for chronic conditions in primary care: an integrative review
Source: BMC Prim Care. 2022 Jan 14;23:8. doi: 10.1186/s12875-021-01595-6 (PMC8759162; doi:10.1186/s12875-021-01595-6)
Supplement: Supplementary file 4 — Additional file 4. Mixed Method Appraisal Tool of Included Studies. [file 12875_2021_1595_MOESM4_ESM.docx]

Additional file 4: Mixed Method Appraisal Tool of included studies

| **Study** | **Methodological Quality Criteria** | | | | | **Comments** |
| --- | --- | --- | --- | --- | --- | --- |
| **Qualitative** | **1.1** | **1.2** | **1.3** | **1.4** | **1.5** |  |
| Abdulrhim, 2021 | Y | Y | Y | Y | Y |  |
| Banfield, 2017 | Can’t tell | Y | Y | Y | Y |  |
| Burridge, 2016 | Y | Y | Y | Y | Y |  |
| Burridge, 2017 | Y | Y | Y | Y | Y |  |
| Davis, 2018 | Y | Y | Y | Y | Y |  |
| Foster, 2015 | Y | Y | Y | Y | Y |  |
| Fu, 2018 | Y | Y | Y | Y | Y |  |
| Grohmann, 2017 | Y | N | Y | Y | Y |  |
| Hannane, 2019 | Y | Y | Y | N | Y |  |
| Hepworth, 2013 | Y | Y | Y | Y | Y |  |
| Hudon, 2016 | Y | Y | Y | Y | Y |  |
| Jones, 2011 | Y | Y | N | N | N |  |
| Karlsson, 2015 | Y | N | Y | Y | Y | 1.2 – two focus groups per country - 8 different European countries with different healthcare systems) |
| Klarare, 2017 | Y | Y | Y | Y | Y |  |
| Knowles, 2015 | Y | Y | Y | Y | Y |  |
| MacPhail, 2009 | Y | Y | Y | Y | Y |  |
| McDonald, 2012 | Y | N | Y | N | N | Query whether the case study approach was most appropriate to the research question and unclear whether data saturation was reached. |
| Penney, 2013 | Y | Y | N | N | Y | 1.3 & 1.4 – too few quotes provided |
| Pullon, 2011 | Y | N | Y | Can’t tell | Y | 1.2 – qualitative approach may not be most suitable to test feasibility.  1.4 – very small sample size (n=4). |
| Purcell, 2019 | Y | Y | Y | Y | Y |  |
| Quigley, 2021 | Y | Y | Y | N | N | 1.4/1.5 – large sections of quotes with minimal interpretation and description in the results section. |
| Roberge, 2016 | Y | Y | Y | Y | Y | 1.2 – the three different clinics may differ in patient groups |
| Ryan, 2020 | Y | Y | Y | Y | Y | Theme labels are not adequately descriptive and are more reflective of categories. |
| Ryrie, 2001 | Y | Y | Can’t tell | N | N | 1.4 – No exemplar quotes provided |
| Simpson, 2008 | Y | Y | Y | N | N |  |
| Soderberg, 2015 | Y | Y | Y | Y | Y | Table with exemplar quotes contributes to adequate demonstration of data. |
| Sorensen, 2020 | Y | Y | Y | Y | Can’t tell | 1.5 Shortest interview was 14 minutes – unsure if enough time to obtain in-depth responses |
| Sundstrom, 2017 | Y | Y | Y | Y | Y |  |
| Talabani, 2017 | Y | Y | Y | Y | Y |  |
| Tan, 2013 | Y | Y | Can’t tell | Can’t tell | Y | 1.3 & 1.4 – small amount of data from patients (exemplar quotes) |
| Taylor, 2018 | Y | Y | Y | Y | Y |  |
| Van Dongen, 2017 | Y | Y | Y | Y | Y |  |
| Walker, 2013 | Y | Y | Y | Y | N |  |
| Wilson, 2018 | Y | Y | N | N | Y |  |
| **Quantitative Randomized Controlled Trials** | **2.1** | **2.2** | **2.3** | **2.4** | **2.5** |  |
| Miller-Rosales, 2020 | Can’t tell | Y | N | N | Y | 2.1 – inadequate description of randomisation  2.3 - <50% response rate  2.4 – Assessors are aware of the group allocation |
| **Quantitative Non-Randomized Study** | **3.1** | **3.2** | **3.3** | **3.4** | **3.5** |  |
| Gorina, 2013 | Y | Y | Y | N | Y |  |
| Tiozzo, 2019 | Y | Y | Can’t tell | N | Y | 3.4 – numerous potential confounders |
| **Quantitative Descriptive** | **4.1** | **4.2** | **4.3** | **4.4** | **4.5** |  |
| Banfield, 2019 | Y | N | Can’t tell | N | Can’t tell | 4.3 and 4.5 – Unsure as these are inadequately reported in paper |
| Ede, 2015 | Y | N | Y | N | Y |  |
| Maeng, 2013 | Y | N | Y | N | Y | 4.2 – unclear as did not test, 4.4 – Higher likelihood for the comparison cohort |
| **Mixed-Methods** | **5.1** | **5.2** | **5.3** | **5.4** | **5.5** |  |
| Balasubramanian,  2017 | Y | Y | Can’t tell | Y | Y |  |
| Butters, 1993 | Y | Y | N | Can’t tell | N |  |
| Drainoni, 2014 | Y | Y | N | Y | N |  |
| Freyens, 2006 | Y | N | N | N | N |  |
| Grimmer-Somers, 2010 | Y | N | Y | Can’t tell | N |  |
| Grol, 2020 | N | Y | Y | Can’t tell | N | 5.1 Explanation or rationale was not provided for MM design |
| Otero-Sabogal, 2010 | Y | Y | Y | Can’t tell | Can’t tell | 5.4 – inconsistencies between quant and qual were not the purpose of mixed methods approach  5.5 – Too little data to adequately answer this question |
| Reiss-Brennan, 2014 | N | N | N | Y | Y | 5.1 – Authors’ own interpretations on different clinics, 5.2 – sample representation of target population not examined, |

Methodological quality criteria as per: Hong QN, Pluye P, Fàbregues S, Bartlett G, Boardman F, Cargo M, Dagenais P, Gagnon M-P, Griffiths F, Nicolau B, O’Cathain A, Rousseau M-C, Vedel I. Mixed Methods Appraisal Tool (MMAT), version 2018. Registration of Copyright (#1148552), Canadian Intellectual Property Office, Industry Canada.

| Category of study designs | Methodological quality criteria |
| --- | --- |
| 1. Qualitative | 1.1. Is the qualitative approach appropriate to answer the research question? |
|  | 1.2. Are the qualitative data collection methods adequate to address the research question? |
|  | 1.3. Are the findings adequately derived from the data? |
|  | 1.4. Is the interpretation of results sufficiently substantiated by data? |
|  | 1.5. Is there coherence between qualitative data sources, collection, analysis and interpretation? |
| 2. Quantitative  randomized controlled  trials | 2.1. Is randomization appropriately performed? |
|  | 2.2. Are the groups comparable at baseline? |
|  | 2.3. Are there complete outcome data? |
|  | 2.4. Are outcome assessors blinded to the intervention provided? |
|  | 2.5 Did the participants adhere to the assigned intervention? |
| 3. Quantitative nonrandomized | 3.1. Are the participants representative of the target population? |
|  | 3.2. Are measurements appropriate regarding both the outcome and intervention (or exposure)? |
|  | 3.3. Are there complete outcome data? |
|  | 3.4. Are the confounders accounted for in the design and analysis? |
|  | 3.5. During the study period, is the intervention administered (or exposure occurred) as intended? |
| 4. Quantitative  descriptive | 4.1. Is the sampling strategy relevant to address the research question? |
|  | 4.2. Is the sample representative of the target population? |
|  | 4.3. Are the measurements appropriate? |
|  | 4.4. Is the risk of nonresponse bias low? |
|  | 4.5. Is the statistical analysis appropriate to answer the research question? |
| 5. Mixed methods | 5.1. Is there an adequate rationale for using a mixed methods design to address the research question? |
|  | 5.2. Are the different components of the study effectively integrated to answer the research question? |
|  | 5.3. Are the outputs of the integration of qualitative and quantitative components adequately interpreted? |
|  | 5.4. Are divergences and inconsistencies between quantitative and qualitative results adequately addressed? |
|  | 5.5. Do the different components of the study adhere to the quality criteria of each tradition of the methods involved? |
